# Role of m6A Methylation in the Occurrence and Development of Heart Failure

**DOI:** 10.3389/fcvm.2022.892113

**Published:** 2022-06-24

**Authors:** Shaowei Fan, Yuanhui Hu

**Affiliations:** Guang’anmen Hospital, China Academy of Chinese Medical Sciences, Beijing, China

**Keywords:** m6A methylation, heart failure, cardiovascular disease, calcium homeostasis, autophagy

## Abstract

N6-methyladenosine (m6A) RNA methylation is one of the most common epigenetic modifications in RNA nucleotides. It is known that m6A methylation is involved in regulation, including gene expression, homeostasis, mRNA stability and other biological processes, affecting metabolism and a variety of biochemical regulation processes, and affecting the occurrence and development of a variety of diseases. Cardiovascular disease has high morbidity, disability rate and mortality in the world, of which heart failure is the final stage. Deeper understanding of the potential molecular mechanism of heart failure and exploring more effective treatment strategies will bring good news to the sick population. At present, m6A methylation is the latest research direction, which reveals some potential links between epigenetics and pathogenesis of heart failure. And m6A methylation will bring new directions and ideas for the prevention, diagnosis and treatment of heart failure. The purpose of this paper is to review the physiological and pathological mechanisms of m6A methylation that may be involved in cardiac remodeling in heart failure, so as to explain the possible role of m6A methylation in the occurrence and development of heart failure. And we hope to help m6A methylation obtain more in-depth research in the occurrence and development of heart failure.

## Background

M6A methylation is the process of methylation modification on the nitrogen atom of adenine in RNA molecule, that is, the methyl provided by S-adenosylmethionine (SAM) is added to the N6 site of adenosine (1). M6A methylation is the most abundant mRNA modification in eukaryotes and is reversible. In addition, m6A methylation is involved in post transcriptional modification. There are about 3–5 m6As in each mRNA, and there are mainly two binding forms of m6A methylation, GC and AC, with GC accounting for about 70% and AC accounting for about 30%. The location of m6A is mainly near the stop codon, internal long exon and 3’untranslated region (3′ UTR) (2–4). M6A methylation, which accounts for only 0.2–0.6% of the total adenosine of mRNA, is the most abundant internal chemical modification in RNA (5, 6). It plays an important role in regulating mRNA processing and metabolism (6–8), including the processing of mRNA precursors (9–11), mRNA output (12), mRNA stability (13), and translation (14–16), and also plays a significant role in the modification of a large number of non-coding RNAs, such as tRNA, rRNA, snRNA, etc., which affect the regulation of genetic information and ultimately development in stress response, immunity and disease (6, 17). In addition, m6A methylation can also exist in precursor RNA (pre-RNA). Methyltransferase is mainly responsible to act as a Writer (such as METTL3/14 RNA methyltransferase complex) in the regulation of m6A (18, 19). Demethylase acts as an Eraser (such as ALKBH515 and FTO) (20, 21). The downstream function of m6A is mediated by Reader proteins that recognize m6A and regulate mRNA processing, such as YTH domain family proteins and IGF2BP1-3 (9, 13, 22, 23), which finally transmit signals to the downstream and trigger downstream biological effects, such as promoting RNA transcription, splicing nuclear output, stability and translation (24).

Heart failure is a complex cardiovascular syndrome, which often occurs in the final stage of a variety of cardiovascular diseases, or secondary to other disease states. The core of its occurrence and development is the structural or functional abnormalities caused by acute or chronic injury, resulting in ventricular filling disorder or abnormal cardiac output (25, 26). Clinically, left ventricular ejection fraction (LVEF) is often used to classify, evaluate the condition and judge the prognosis, therapeutic effect and carry out clinical related research. According to different ejection fraction (EF), different treatment schemes and even rescue measures are determined. According to the heart failure guidelines in 2022 (25), heart failure is divided into four categories according to different levels of LVEF: (1) HFrEF (HF with reduced EF) is defined as LVEF ≤40%; (2) HFimpEF (HF with improved EF) is defined as previous LVEF ≤40%, and subsequent measurement LVEF >40%; (3) HFmrEF (HF with mildly reduced EF) is defined as LVEF 41–49%, accompanied by elevated left ventricular filling pressure, such as elevated natriuretic peptide or hemodynamic measurement results; (4) HFpEF (HF with preserved EF) is defined as LVEF ≥ 50%, accompanied by elevated left ventricular filling pressure. The characteristics of HFpEF are: most of them are accompanied by different degrees of inflammatory and metabolic complications and chronic comorbidities, such as obesity, hypertension, diabetes mellitus type 2, renal insufficiency, to name a few; changes in cardiac structure and cardiomyocytes, such as cardiomyocyte hypertrophy, fibrosis and inflammation; it mainly affects left ventricular diastolic function and reduces compliance (27); endothelial dysfunction and microvascular complications appeared earlier and more common (28, 29). HFrEF is characterized by acute or chronic loss of a large number of cardiomyocytes due to myocardial ischemia and myocarditis; it mainly affects left ventricular systolic function (30, 31). HFmrEF and HFimpEF is the transitional stages between the two. It is easy to progress to HFrEF, but the overall prognosis of HFrEF is better (32). According to the global burden of diseases, injuries, and risk factors study 2017 (GBD 2017), the number of patients with heart failure worldwide has exceeded 6.4 million (33). With the increase of population growth and aging, the health burden caused by heart failure has been similar to the combined incidence rate of lung cancer, breast cancer and prostate cancer (34). People with hypertension, obesity, kidney disease or ventricular systolic dysfunction before the age of 35 may have related manifestations of heart failure after 20 years at the latest (35). In addition, patients with heart failure had a higher incidence of sudden cardiac death (36). What’s more, in the past 20 years, the morbidity of heart failure with preserved ejection fraction has increased, and its morbidity has increased significantly in both men and women over time (34, 37). By 2030, the morbidity of heart failure is expected to rise by 46%, affecting more than 8 million people (38). Therefore, more attention should be paid to heart failure. The possible pathogenesis and effective treatment targets of heart failure will become the focus of research for a long time in the future.

The upsurge of m6A methylation research may bring new directions and new ideas for the in-depth study of the pathogenesis of heart failure. Targeted regulation of m6A methylation related processes may contribute to the diagnosis, treatment and drug development of patients with heart failure. Therefore, in this review, we summarize the possible mechanism of m6A methylation in the occurrence and development of heart failure, in order to illustrate the importance of exploring the role of m6A.

## M6A Methylation

**FIGURE 1:**
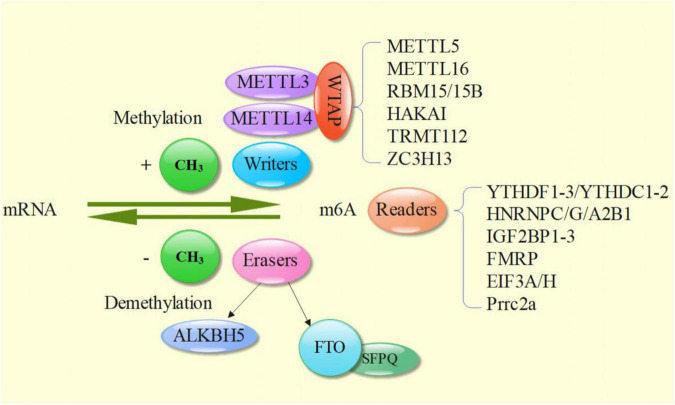
Reversible process of m6A modification and three factors - Writer, Eraser, and Reader.

M6A methylation needs three main parts to complete together, namely the Writers, the Erasers, the Readers (Figure 1).

### M6A Writers

M6A Writer is completed by m6A methyltransferase complex, namely methyltransferase like 3 (METTL3) and METTL14 and their cofactor Wilms tumor 1 associated protein (WTAP). METTL3 contains S-adenosylmethionine (SAM) binding domain and DPPW motif (Asn-Pro-Trp), which can transfer methyl from SAM to N6 position of target adenosine (18, 39); METTL14 supports METTL3 structurally by providing RNA binding scaffolds, which greatly improves the methylation efficiency (24). In addition, although WTAP does not have direct catalytic activity, it participates in the localization of METTL3-METTL14 heterodimer in nuclear spots and promotes the accumulation of m6A (40). Finally, a ternary METTL3: METTL14: WTAP complex was formed and relocated to the nuclear spot (18, 41). VIRMA (protein virilizer homolog or called KIAA1429) mediates the preferential methylation of m6A mRNA in 3′ UTR and near termination codon, plays an essential role in guiding regional selective methylation, and can help METTL3 and METTL14 locate in nuclear plaque (42). In addition, HAKAI, zinc finger CCCH type containing 13 protein (ZC3H13) and RNA binding motif protein 15 (RBM15) have been shown to participate in and improve the abundance of mRNA m6A (43–45). In the process of m6A methylation of other RNA types, ZC3H13, RBM15/15b, METTL5, tRNA methyltransferase homologue 112 (TRMTL112), METTL16, and other methyltransferases play important roles, as well (46–48).

### M6A Erasers

M6A eraser is m6A demethylase, which includes the currently known ALKB family member 5 (ALKBH515) and fat mass and obesity associated protein (FTO), revealing the reversibility of RNA modification. The demethylation activity of FTO makes contribution to the normal development of human central nervous system and cardiovascular system (20, 49). The m6A demethylation of FTO requires two-step oxidation reaction: firstly, m6A is oxidized to N6 hydroxymethyladenosine (hm6A), and then hm6a is oxidized to N6 formyl adenosine (f6A), which can be converted into two stable substances: formaldehyde and formic acid, and the final product is adenosine (50). Multifunctional nucleoprotein - splicing factor rich in proline and glutamine (SFPQ)—can directly interact with FTO, promote m6A demethylation effect, and participate in a variety of cell activities, including RNA transport, apoptosis and DNA repair (51, 52). ALKBH5 can exert oxidative demethylation effect *in vivo* and *in vitro*, and co-locate with nuclear spots to complete RNA regulation (53).

### M6A Readers

M6A Reader binding sites are overlapped with m6A localization, both near the stop codon of CDS and 3 ′ UTR on mRNA (54). At present, the YT521-B homology (YTH) family of proteins is the most widely studied in m6A Readers. Its main feature is that it has YT domain that can stably recognize m6A, including YTHDF1, YTHDF2, YTHDF3, YTHDC1 and YTHDC2 (55). YTHDF plays a synergistic role in regulating mRNA transcription and translation (56). In addition, there are heterogenetic nuclear ribonucleoproteins (hnRNPs), which can combine with some structures formed by m6A reconstructed partial RNA to regulate transcription and translation. Members include hnRNPC, hnRNPG and hnRNPA2B1. In addition, there are readers containing the same RNA binding domains (RBD), such as insulin like growth factor 2 mRNA binding proteins 1–3 (IGF2BP1-3), frame X mental retention protein (FMRP), EIF3A/H and Prrc2a (57). EIF3A/H is preferentially crosslinked with m6A containing mRNA rather than unmethylated RNA (58). Prrc2a is a recently discovered reader, and the specific mechanism is unknown (57).

## Relationship Between M6A and Heart Failure

The relationship between m6A methylation and heart failure has gradually attracted people’s attention with the deepening exploration of research. M6A is maladjusted in heart failure and plays a key role in cardiovascular diseases, such as ventricular septal and atrioventricular defects, hypertrophic cardiomyopathy, arrhythmia, coronary heart disease, ischemic heart failure and so on (49, 59–61). For example, up regulation of m6A methylation can promote compensatory myocardial hypertrophy, while down regulation is related to eccentric cardiomyocyte remodeling and dysfunction (62). As an m6A Eraser, the FTO protein expression is reduced, which can make more m6A complete transcription and reduce the contractility of cardiomyocytes (59). The overexpression of FTO can enhance myocardial contractility, improve cardiac function and delay the development of heart failure by demethylating contraction genes, such as, sarcoplasmic reticulum (SR) -Ca^2+^ ATPase 2a (SERCA2a), ryanodine 2 (RyR2), myosin heavy chain (MYH) 6/7, and increasing their protein expression level, and even reversing cardiac fibrosis and inducing angiogenesis in heart failure after myocardial infarction. M6A methylase METTL3 can promote cardiomyocyte hypertrophy *in vitro* and *in vivo*. And increasing m6A methylation will promote compensatory cardiomyocyte hypertrophy, while reducing m6A methylation will induce eccentric cardiomyocyte remodeling and dysfunction (63). The level of m6A in dilated cardiomyopathy patients with heart failure is higher than that in patients without heart failure (64). Therefore, m6A methylation is closely related to the occurrence and development of heart failure.

## Possible Mechanism of M6A Methylation in the Occurrence and Development of Heart Failure

**FIGURE 2:**
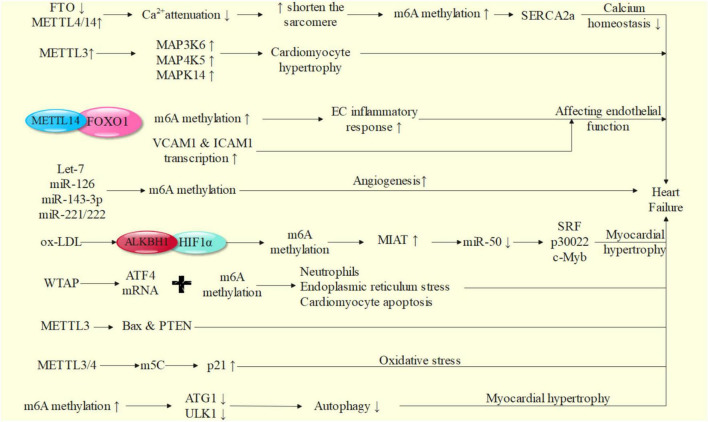
Possible mechanism of m6A methylation in heart failure.

We will explore the possible mechanism of m6A in the occurrence and development of heart failure from the aspects of calcium homeostasis, inflammatory response, autophagy, oxidative stress, neurohumoral regulation, vascular endothelial dysfunction, renin angiotensin aldosterone system activation and so on (65) (Figure 2).

### m6A Methylation and Calcium Homeostasis

Dysregulation of calcium homeostasis in cardiomyocytes is considered to be one of the main causes of heart failure. It not only affects the systolic and diastolic function of cardiomyocytes (i.e., the decline of cardiac pump function), but also affects the transmission of ECG signals (i.e., arrhythmia) and myocardial apoptosis and hypertrophy.

Under normal circumstances, myocardial contraction is completed through excitation contraction coupling. After the action potential is transmitted to the cardiomyocyte membrane and enters the T-tube depression, it triggers the opening of L-type calcium channel (LTCC), and some calcium ions flow into cardiomyocytes, acting on the calcium ion channel of SR, triggering the release of a large amount of calcium ions from sarcoplasmic reticulum, so that the calcium ion level in cardiomyocytes quickly reaches the peak, that is, calcium ion spark. The sum of time and space of calcium ion sparks is called calcium ion transient. Subsequently, calcium ions bind to troponin on the fine myofilament, causing changes in the spatial conformation of the fine myofilament, exposing the binding sites of actin and myosin on the thick myofilament, and the two bind to complete a myocardial contraction. However, when heart failure occurs, on the one hand, due to the reduction of SERCA2a function, the increase of RyR leakage, the increase of Na^+^-Ca^2+^ exchange (NCX) function competing with SERCA2a for Ca^2+^, the Ca^2+^ released by SR after LTCC activation is reduced, which can not meet the transient of calcium ions in cardiomyocytes, resulting in the significant decrease of myocardial contractility (66). On the other hand, cardiomyocyte hypertrophy causes the prolongation of action potential and affects the synchronous release of calcium ions (67). This is the mechanism of HFrEF or systolic heart failure. After myocardial contraction, the calcium concentration in the sarcoplasma is rapidly reduced through SERCA2a and plasma membrane NCX pump, so that the thick and thin muscle filaments are dissociated after restoring the original conformation, and finally complete cardiac relaxation (68). However, failing cardiomyocytes often can not ensure the coordination and rapidity of diastolic process, which may lead to normal ventricular systolic function and abnormal diastolic filling (69–71). This is HFpEF or diastolic heart failure.

In the process of heart failure, the expression of m6A demethylase FTO in cardiomyocytes decreases, the expression of writing proteins such as METTL4/14 increases, and the content of m6A in failed heart and hypoxic cardiomyocytes increases (59, 72). By affecting the transcriptional expression of contractile protein SERCA2a, it leads to abnormal regulation of calcium homeostasis, so as to reduce myocardial contractile function. *In vivo* and *in vitro* studies show that FTO can improve the amplitude of Ca^2+^, accelerate the attenuation of Ca^2+^, shorten the sarcomere, reduce the increase of m6A induced by ischemia, and improve the corresponding systolic dysfunction of cardiomyocytes. After enrichment and screening of pathways, FTO can selectively act on pathways related to cardiac sarcomere tissue, myofibril assembly, calcium treatment and contraction, and can cause various heart diseases, such as hypertrophic cardiomyopathy, ventricular septal defect, atrioventricular defect, arrhythmia and coronary heart disease (59, 61). Cardiomyocyte hypertrophy is accompanied by the increase of m6A methylation level. In this process, the overexpression of m6A RNA methylase METTL3 plays an important role in increasing the levels of mitogen activated protein (MAP) 3K6, MAP4K5 and MAPK14, which leads to cardiomyocyte hypertrophy (62, 63). *In vitro* experiments confirmed that inhibition of METTL3 could effectively block cardiomyocyte hypertrophy (73–75). Nearly a quarter of transcripts in human heart are related to m6A methylation, and FTO gene knockout can lead to impaired cardiac function (76).

In conclusion, m6A methylation may play a promoting role in the occurrence and development of heart failure by affecting calcium regulation related genes, affecting myocardial calcium homeostasis, myocardial contractility, cardiac contraction and relaxation.

### m6A Methylation and Inflammatory Response

Vascular endothelial cells play an important role in cardiovascular diseases and normal physiological metabolism, such as participating in angiogenesis, promoting wound healing, inducing smooth muscle cell proliferation and fibrosis, and reactive oxygen species (ROS) participate in vascular inflammatory response. Among them, inflammatory response has always been the key point of cardiovascular disease research. ROS plays a significant role in inflammatory response.

Endothelial cells are the main cell type involved in coordinating the pathological changes of heart failure, and their surface is often the direct place of a variety of inflammatory reactions (75). During the occurrence and development of heart failure, chronic inflammatory reaction can continuously promote the abnormal changes of myocardial structure and function (71, 77). Therefore, a variety of pro-inflammatory factors on the surface of vascular endothelial cells are closely related to the occurrence and development of heart failure. For example, VCAM1, an adhesion molecule activated on the surface of endothelium, can promote leukocyte adhesion and epithelial cell migration and trigger intravascular inflammatory response by binding with ligands on leukocyte membrane (78, 79). VCAM1 can promote the production of ROS and promote the occurrence of heart failure by activating matrix metalloproteinase to cause ventricular remodeling (80). ROS can directly damage the cell membrane, induce cardiomyocyte apoptosis, cause damage to myocardial systolic and diastolic function, reduce cardiac output, increase ventricular filling pressure, cause ventricular dilation and ventricular remodeling, and promote the further development of heart failure. VCAM1 can promote the differentiation and infiltration of immune cells, which is positively correlated with the risk of heart failure and promote the development of heart failure (81–83). In addition, T lymphocytes (mainly T1 cells) can infiltrate into myocardial tissue, possibly through the production of interferon- α, transforming myocardial fibroblasts to γ-smooth muscle actin fibroblast (84). The transformation of γ-smooth muscle actin fibroblasts further causes the proliferation of myocardial fibroblasts, leads to ventricular dilation and intensifies ventricular remodeling, which may also be related to the occurrence and development of heart failure (85). M6A methylase METTL14 can bind to FOXO1 and mediate its m6A methylation to induce the inflammatory response of endothelial cells (EC) (86). FOXO1 is an important transcription factor, which can promote the promotion of transcription and affect the occurrence and development of heart failure by directly acting on the promoter regions of VCAM1 and intercellular adhesion molecule-1 (ICAM1).

As we all know, cardiac hypertrophy is the main sign of heart failure. MiRNA molecules, such as non-coding RNA members, can also play an essential role in the pathological changes of heart failure by mediating myocardial hypertrophy, and can be modified by m6A (87). For example, Let-7, miR-126, miR-143-3p and miR-221/222 can affect the function of endothelial cells and vascular smooth muscle cells in angiogenesis. Let-7, miR-25 and miR-375 have been proved to play key roles in the pathogenesis of a variety of cardiovascular diseases, and participate in the regulation of apoptosis, autophagy, oxidative stress, inflammatory response and calcium treatment, so as to participate in the occurrence of heart failure (88–91). Therefore, the study of m6A methylation of these molecules is particularly important, which may help to further explore the pathogenesis of heart failure.

### M6A Methylation and Autophagy

As we all know, during the occurrence and development of heart failure, with the activation of nerve body fluid and the increase of hemodynamic load, myocardial compensatory hypertrophy increases the volume of cardiomyocytes and the number of mitochondria, so as to maintain close to normal cardiac output and supply the needs of the body and tissue cells. However, when the myocardium is irreversibly and continuously hypertrophic, the cardiomyocytes are gradually in the state of ischemia and hypoxia, and can not bear the continuous pressure load, decompensated heart failure eventually occurring.

Autophagy is a process in which eukaryotic cells play a degradation function through lysosomes to inactivate and phagocytize proteins and cell components. It plays an important role in normal physiological function, proliferation, death and maintaining intracellular homeostasis. The main processes include: forming phagocytic vesicles of phospholipid bilayer, phagocytizing and wrapping damaged organelles and abnormally expressed proteins to form autophagosomes, which are the landmark products of autophagy; then, the vesicles are transported to the lysosome and combined with the lysosome to form autophagic lysosomes, which are finally degraded (92, 93). In mammalian cells, autophagy currently known includes at least three different pathways: macro-autophagy, micro-autophagy and chaperone mediated autophagy (CMA) (94, 95). Among them, the difference among the three mainly lies in the process of binding with lysosomes: macro-autophagy is that cytoplasmic components are directly attached to lysosomes and then directly degraded by lysosomal hydrolases; Micro-autophagy is the invagination of lysosomal membrane, which is engulfed by lysosomes after forming vesicles; CMA enters the lysosome through lysosomal associated membrane protein 2A (LAMP-2A) receptor in the form of complex with the help of chaperone proteins such as Hsc-70 (96, 97).

Autophagy plays a vital role in the occurrence and development of heart failure guided by myocardial hypertrophy (98, 99). Some biomolecules promote autophagy, which helps to inhibit myocardial hypertrophy and delay the process of heart failure, such as cathepsin-L and activated protein kinase (AMPK) (100). AMPK can activate T cell nuclear factor (NFAT), MAPK, nuclear factor kappa B (NF-κB), FOXO and MuRF1 to antagonize myocardial hypertrophy caused by increased pressure load and RAS system (101–103). Over activation or inactivation of autophagy induced by some biomolecules will promote myocardial hypertrophy and pathological remodeling, and worsen the progression and condition of heart failure (104–106), such as overexpression of autophagy promoting protein Beclin-1 (106), the family of Toll like receptors 3 (TLR3) (94), METTL3 (64, 74), to name a few. In addition, autophagy can also occur in the process of heart failure caused by a variety of cardiovascular diseases, such as ischemic heart disease, dilated cardiomyopathy and heart valve disease (104, 105). AMPK can promote autophagy by activating mammalian target of rapamycin (mTOR) C1 and activate mTORC2 to avoid excessive autophagy activity, which is contrary to each other to delay the process of heart failure (99). During ischemia-reperfusion, researchers observed increased autophagy formation, which may be related to AMPK (107). Transcription factor EB (TFEB) is a key regulator of autophagy (108). Disaccharide trehalose can up regulate TFEB, promote autophagy and inhibit cardiomyocyte apoptosis (109). At present, the contradiction of relevant research is that m6A methylation may be affected by a variety of additional microenvironment factors in different heart diseases, resulting in changes in the behavior of methylated transcripts, such as excessive pressure load, hypoxia and so on (110). M6A transcripts in the heart of aortic coarctation (TAC) mice are reduced compared with the sham operation group, which is related to the reduced expression of methylase METTL3 (64). Therefore, autophagy has been proved to be closely related to the occurrence and development of heart failure, but a lot of in-depth research is still needed to explore its mechanism.

M6A methylation can affect the expression of autophagy related genes, regulate autophagy, and play an important role in the occurrence and development of heart failure (97, 111, 112). It is reported that m6A methylation can inhibit autophagy by affecting the post transcriptional regulation of autophagy related gene 1 (ATG1)/Unc-51-like kinase 1 (ULK1) (113). Similarly, it can inhibit autophagy by affecting autophagosome formation (114). M6A demethylase FTO can up regulate ULK1 protein and promote autophagy related protein expression (113), indicating that FTO actively regulates autophagy in an enzyme activity dependent manner, thus playing a key role in cardiac remodeling and rehabilitation (59).

In conclusion, m6A methylation may affect myocardial hypertrophy, cardiac remodeling and promote the occurrence and development of heart failure by regulating autophagy.

### M6A Methylation and Ischemia and Hypoxia

M6A methylation is involved in regulating mRNA stability, protein expression and various physiological reactions of cells. It is also a key molecule in the process of affecting the contractile function of cardiomyocytes due to ischemia and hypoxia, which can cause ischemic myocardial damage. This is the main cause of cardiovascular diseases including myocardial infarction and heart failure, so it also deserves attention.

LncRNA myocardial infarction associated transcript (MIAT) is a known hypoxia response gene. The increase of MIAT expression level was detected in the whole blood of patients with non-ST segment elevation myocardial infarction (NSTEMI) and ST segment elevation myocardial infarction (STEMI) (115, 116). Similarly, in myocardial hypertrophy induced by angiotensin II, MIAT increased significantly. By inhibiting miR-150 and then affecting the expression of serum response factor (SRF) (117), p30022 and c-Myb (118), MIAT promotes cardiomyocyte hypertrophy and fibroblast activation, and finally promotes the development of myocardial hypertrophy (119). Oxidized low density lipoprotein (ox-LDL) induces m6A demethylation in hypoxia inducible factor 1α (HIF1α) binding region through ALKBH1, which can promote MIAT transcriptional activation, become a key factor in myocardial infarction and myocardial hypertrophy, and affect myocardial contractility (120). WTAP has been proved to regulate the m6A modification of activating transcription factor 4 (ATF4) mRNA, promote ischemic myocardial injury by promoting neutrophil infiltration, endoplasmic reticulum stress and cardiomyocyte apoptosis, and finally lead to cardiac dysfunction and heart failure (121, 122). M6A methylation mediated by METTL3 shows different effects. When the factors inducing myocardial hypertrophy appeared, the expression of METTL3 mediated m6A methylation increases significantly, which promotes the occurrence of compensatory myocardial hypertrophy; when m6A methylation expression mediated by METTL3 is reduced and the observation time is long enough, it can lead to eccentric cardiomyocyte remodeling and cardiac dysfunction (62). B cell lymphoma-2 (BCL2) -associated X protein (Bax) and gene of phosphate and tension homology deleted on chromosome ten (PTEN) are target genes downstream of METTL3 under hypoxia/reperfusion injury of cardiomyocytes *in vitro*, which can cause myocardial damage (123).

Therefore, in the future, the research on FTO and METTL3 related myocardial ischemia and hypoxia may find a breakthrough for the therapeutic target of heart failure.

### M6A Methylation and Oxidative Stress

Oxidative stress is a common reaction of cell aging and affecting cell function. When the balance between oxidants and antioxidants is broken, it will lead to oxidative stress. Among them, a variety of oxides mainly play an essential role, such as ROS, RON (a kind of tyrosine kinase receptor) and so forth. These substances will affect the normal replication, transcription and translation of genetic substances, such as DNA, and can affect the normal function of cardiomyocytes, resulting in cardiomyocyte damage and promoting the occurrence and development of heart failure.

METTL3/METTL4 may eventually improve the translation level of p21 by promoting other forms of mRNA methylation (like m5C), so as to affect its expression in the process of oxidative stress and cell aging, promote the accumulation of oxides such as ROS and start the oxidative stress response (124). Therefore, it is difficult to make a great breakthrough in the research of m6A methylation in oxidative stress of heart failure cardiomyocytes, which may be due to the existence of various forms of cross-linking in mRNA methylation itself.

## Perspectives

**FIGURE 3:**
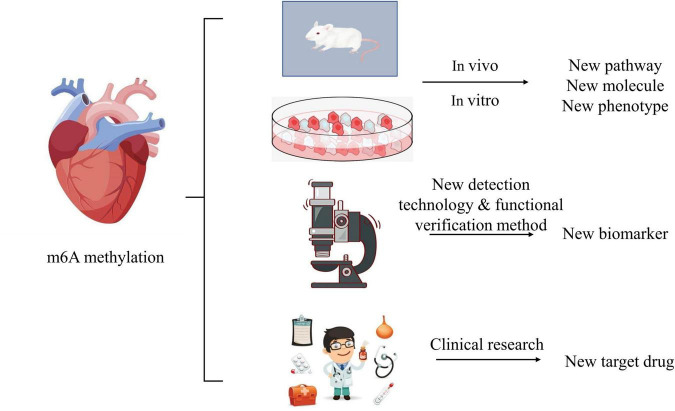
The flow-chart of further research in the field of heart failure.

The role of m6A methylation in the mechanism of heart failure is still unknown: on the one hand, it is necessary to build heart failure models with the help of more animals and cells to explore the relationship between m6A methylation and known mechanisms, pathways and molecules that may be involved. Gradually expanding and exploring the regulatory process of m6A methylation, such as the upstream and downstream regulatory pathway of m6A methylation, count a lot. This may improve the understanding of m6A methylation in the pathogenesis of heart failure and bring new breakthroughs. On the other hand, it is necessary to improve the detection technology and functional verification method of m6A methylation, and to increase bioinformatics analysis to find new biomarkers of m6A methylation involved in the occurrence and development of heart failure. In addition, the role of m6A methylation in multiple systems and multiple organs is still unclear in the clinical study of the prevention and treatment of heart failure. With the deepening exploration of research, it needs the efforts of several generations to explore whether we can realize the targeted treatment scheme and drug research and development like tumor targeted drugs in the future, simplify the dosage of drugs, reduce the side effects of drugs, improve the quality of life of patients, and reduce the mortality and disability rate. We still have a long way to go in the treatment and prevention of heart failure (Figure 3).

## Conclusion

In conclusion, m6A methylation, as one of the most abundant types of RNA modification, has been gradually studied in cardiovascular diseases. This paper reviews the possible mechanisms of m6A methylation in the occurrence and development of heart failure, hoping to summarize the possible role of m6A, so as to lay a foundation for the accurate treatment of heart failure. The main effect of m6A methylation on heart failure is to promote calcium homeostasis, inflammatory response and autophagy, causing cardiomyocyte hypertrophy, myocardial mitochondrial dysfunction, reducing myocardial contractility and cardiac remodeling, resulting in serious consequences. However, there is still a lack of research on the related mechanisms of m6A methylation in the neurohumoral and renin angiotensin aldosterone systems of heart failure, which may provide new ideas for research and new perspectives for treatment.

## Author Contributions

SF: conception, design, and manuscript writing. YH: manuscript revising. Both authors contributed to the article and approved the submitted version.

## Conflict of Interest

The authors declare that the research was conducted in the absence of any commercial or financial relationships that could be construed as a potential conflict of interest.

## Publisher’s Note

All claims expressed in this article are solely those of the authors and do not necessarily represent those of their affiliated organizations, or those of the publisher, the editors and the reviewers. Any product that may be evaluated in this article, or claim that may be made by its manufacturer, is not guaranteed or endorsed by the publisher.
